# MultiSeq-AMR: a modular amplicon-sequencing workflow for rapid detection of bloodstream infection and antimicrobial resistance markers

**DOI:** 10.1099/mgen.0.001383

**Published:** 2025-04-03

**Authors:** Mohammad Saiful Islam Sajib, Katarina Oravcova, Kirstyn Brunker, Paul Everest, Ma Jowina H. Galarion, Manuel Fuentes, Catherine Wilson, Michael E. Murphy, Taya Forde

**Affiliations:** 1 School of Biodiversity, One Health & Veterinary Medicine, University of Glasgow, Glasgow, UK; 2 MRC-University of Glasgow Centre for Virus Research, Glasgow, UK; 3 Department of Microbiology, New Lister Building, Glasgow Royal Infirmary, NHS Greater Glasgow and Clyde, Glasgow, UK; 4 School of Medicine, Dentistry & Nursing, College of Medical, Veterinary & Life Sciences, University of Glasgow, Glasgow, UK

**Keywords:** amplicon sequencing, antimicrobial resistance (AMR), bloodstream infection, cost-effective, MultiSeq-AMR, rapid diagnosis

## Abstract

Bloodstream infections (BSIs) represent a significant global health challenge, and traditional diagnostic methods are suboptimal for timely guiding targeted antibiotic therapy. We introduce MultiSeq-AMR, a rapid and modular nanopore amplicon-sequencing workflow to identify bacterial and fungal species and a comprehensive set of antimicrobial resistance (AMR) genes (*n*=91) from various types of infection sources. We initially benchmarked MultiSeq-AMR using DNA from 16 bacterial and 5 fungal reference strains and accurately identified all species. AMR gene identification exhibited 99.4% categorical agreement (CA: 153/154 prediction) with whole-genome sequencing. Further validation with 33 BACT/ALERT positive samples from suspected BSI cases revealed 100% accuracy for genus and 96.7% for species identification, with 97.4% CA (151/155) for AMR gene prediction. To accelerate microbiological diagnosis, a 6 h culture enrichment step was tested with MultiSeq-AMR using 15 clinically important bacterial species. Of 13 species selected for sequencing, 11 were correctly identified, with 96% CA (59/61 predictions) for AMR gene identification. With only 2 Mbp yield, sequencing identified 93.7% of species and 89.8% AMR genes initially detected with 20–50 Mbp yield/sample. MultiSeq-AMR holds promise for BSI diagnosis, as species/AMR genes could be identified under 5 h of BACT/ALERT positivity and potentially <11 h of sample collection (rapid-enrichment) for a large set of bacterial species. MultiSeq-AMR gene targets can be modified/increased indefinitely to suit user needs. Further research is required to clinically validate MultiSeq-AMR, especially the rapid enrichment method, to assess its utility in a medical setup and in improving patient outcomes in BSI.

**Impact Statement**

Bloodstream infections (BSIs) are among the leading causes of morbidity and mortality worldwide. Traditional diagnostic approaches, such as blood culture, often provide results only after more than 48 h of sample collection, which can delay critical clinical interventions. Our study addresses this gap by developing MultiSeq-AMR, a modular Oxford Nanopore-based amplicon-sequencing workflow that can be utilized to rapidly identify bacterial and fungal species and a large set of clinically important antimicrobial resistance (AMR) markers (*n*=91) for same-day BSI diagnosis. Our validation using clinical samples demonstrates that MultiSeq-AMR can facilitate accurate species and AMR gene detection under 5 h of BACT/ALERT positivity and as quickly as within 11 h of sample collection. MultiSeq-AMR thus offers a promising advancement in clinical diagnostics, enabling rapid detection of pathogens and their resistance determinants. The workflow’s modularity allows for future expansion and adaption to other infections, making it a versatile tool with the potential to substantially improve patient outcomes through timely and targeted antibiotic therapy.

## Data Summary

Raw amplicon-sequencing reads (fastq.gz) analysed and described in this study can be found on the European Nucleotide Archive under the study accession ‘PRJEB82614’. Data available at: https://www.ebi.ac.uk/ena/browser/view/PRJEB82614.

## Introduction

Bloodstream infections (BSIs) continue to be one of the major public health challenges worldwide, leading to high morbidity and mortality rates despite continuous surveillance efforts [1]. The majority of these cases are traditionally attributed to Gram-positive (e.g. *Staphylococcus*, *Streptococcus* and *Enterococcus*) or Gram-negative (e.g. *Enterobacteriaceae* and *Pseudomonas*) bacterial species; however, reports of fungal and viral causes are also on the rise [1,3]. Timely diagnosis and appropriate antimicrobial therapy are crucial for better patient outcomes in BSI, especially for patients developing early signs of sepsis [4]. Identification and antimicrobial susceptibility testing by traditional culture-based methods, with typical time to positivity up to 48–72 h, are not fast enough to guide targeted antibiotic therapy. Therefore, empirical antibiotic treatment plays a significant role in the early stages of clinical management [5]. Though strict adherence to clinical guidelines can lower mortality of hospitalized critically ill patients, individuals infected with resistant pathogens are less likely to receive the appropriate antibiotic treatment initially, which may lead to poor clinical outcomes and contribute to the selection of antimicrobial resistance (AMR) [6]. Therefore, rapid and sensitive diagnostic methods are urgently required to better inform antibiotic regimens and preserve the limited number of critically important antimicrobials [6,9].

Next-generation sequencing (NGS) is a promising alternative for BSI diagnosis, as certain NGS workflows can rapidly identify bacterial species and/or AMR determinants [10,11]. However, one of the biggest challenges of NGS, specifically unbiased metagenomic next-generation sequencing (mNGS) for BSI diagnosis, is the proportion of host DNA compared to the pathogen. Host represents >99.9% of total nucleic acid in blood samples, and therefore, direct mNGS results in reduced analytical sensitivity, increased sequencing cost and time per sample to identify both pathogen and AMR determinants for rapid patient management [12].

To improve performance, several studies have employed targeted amplicon sequencing (TAS) for detecting bacterial/fungal species and/or AMR genes from different types of samples, where host proportions are significantly higher than those of the pathogen [9,13, 14]. While this approach is sensitive, cost-effective and hence shows promise for BSI diagnosis, one of the major limitations of TAS is its lack of comprehensiveness compared to mNGS in identifying AMR determinants. To date, most studies utilizing TAS have included a smaller collection of AMR targets common for a particular species [14,15]. Multiplex AMR sequencing panels with a more comprehensive collection of targets have been described in the literature; however, none of them have been developed/tested for diagnosing BSI in combination with rapid sequencing platforms for deployment in a clinical laboratory setup [16,17].

This study presents MultiSeq-AMR, a rapid, cost-effective and modular nanopore amplicon-sequencing-based workflow that can identify bacterial and fungal species and a large number of clinically important AMR genes from blood or other culture-enriched samples. We initially developed and benchmarked this workflow using extracted genomic DNA from reference bacterial/fungal isolates. Later, this method was validated with BACT/ALERT (an automated blood culture system) positive and negative culture-enriched samples from individuals showing symptoms related to BSI. To accelerate microbiological diagnosis, MultiSeq-AMR was finally tested on rapid culture-enriched whole blood spiked with clinically important bacterial species.

## Methods

### Reference strains, substitute host matrix and patient samples

Sterile whole blood from healthy human volunteers and sheep (E and O Laboratories, Scotland, United Kingdom) collected in tubes containing EDTA was used as an initial substitute for infected blood samples to benchmark MultiSeq-AMR. The list of clinical isolates and American Type Culture Collection (ATCC) strains used in this study for spiking, enrichment and other experiments can be found in Supplementary Material 1 (Sheet: Sample_info_S1), available in the online Supplementary Material. Blood culture positive (*n*=33) and negative (*n*=3) samples from individuals suspected of having BSI were surplus specimens collected initially as a part of routine diagnostic testing.

### Primer design to identify species and AMR genes

**Fig. 1.**
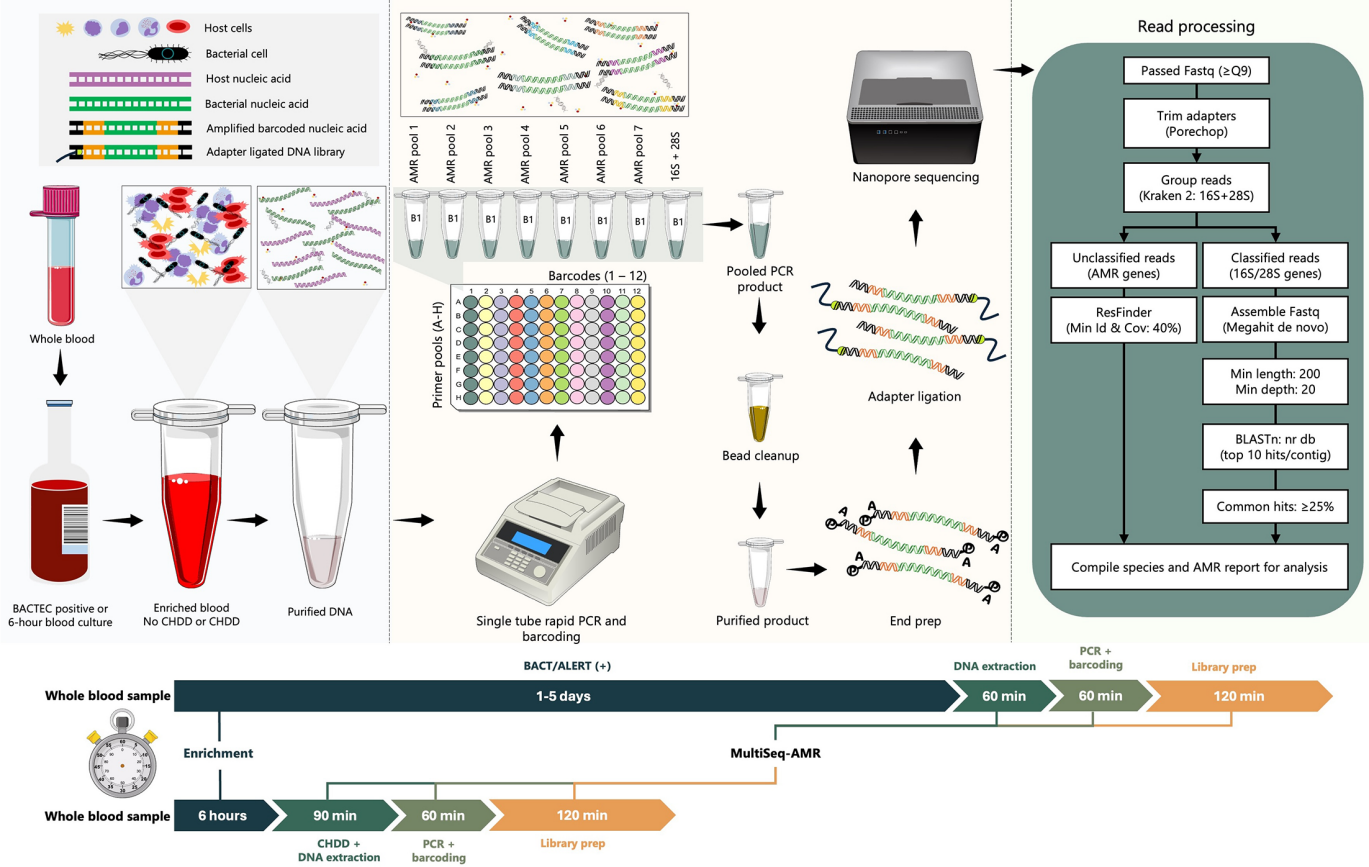
Schematic diagram highlighting major steps in MultiSeq-AMR workflow including DNA extraction, PCR barcoding, library preparation and sequencing and data analysis. Both BACTEC positive and rapid enriched (6 h) blood culture samples can be used, each with slightly different initial processing steps. BACTEC positive samples can be subjected to DNA extraction directly; however, rapid culture-enriched samples must undergo chemical host DNA depletion (CHDD) to reduce host DNA and concentrate bacteria in the sample prior to nucleic acid purification. Total hands-on time for MultiSeq-AMR is 4–4.5 h for 6–8 samples including CHDD and/or DNA extraction, PCR barcoding and library preparation. The PCR plate example shows the arrangement of the seven AMR and one species-specific primer pools (A–H) combined with different barcodes (B1–B12). The first column in the plate (dark teal) highlights barcode 1 (B1).

The current version of MultiSeq-AMR is comprised of 93 targets in a total of 14 primer pools: 1 pool with 2 amplicon targets to identify bacterial or fungal species and 13 clinically important AMR primer pools, each targeting up to 7 unique genes per pool (Fig. 1 and Supplementary Material 2). The species-specific pool contains primers to amplify 16S rRNA [18] and 28S rRNA [19] for bacterial and fungal species identification, respectively. AMR pools include 91 unique gene primers from 11 antibiotic classes (Supplementary Material 1, Sheet: Primer_Seq_S2).

To design MultiSeq-AMR, nucleotide sequences of all the AMR genes and variants were initially obtained from the ResFinder database (bitbucket.org/genomicepidemiology/resfinder_db) (last accessed: 30/07/2024). Each type of AMR gene was aligned using muscle [20] to identify closely related variants and differentiate between groups (e.g. *blaOXA-1*, *blaOXA-9*, *blaOXA-24*, *blaOXA-48*, etc.) for designing primers [20]. Subsequently, National Center for Biotechnology Information (NCBI) Primer-blast software was utilized to design AMR primers with the following parameters: a maximum primer melting temperature (*T*_m_) of 60 °C, no cross-reaction with *Homo sapiens* and a PCR product length between 500 and 800 bp [21]. To differentiate AMR variants with sequencing, variant-specific mutations were kept in the region between the forward and reverse primers for as many AMR targets/variants as possible. For genes/groups of genes where no conserved regions were found using direct Primer-blast, forward/reverse primer sequences were manually developed by incorporating degenerate nucleotide sequences. A total of 82 AMR primer pairs were developed in this study, and the remaining 11 primer pairs (9 AMR targets and 2 targets for species identification) were sourced from previously published studies [22,25]. The 93 primer pairs targeting universal species/AMR genes were then tagged with outer primers having complementary sequences (5′-3′, Forward: TTTCTGTTGGTGCTGATATTGC, Reverse: ACTTGCCTGTCGCTCTATCTTC) for PCR Barcoding Expansion 96 kit [Oxford Nanopore Technologies (ONT), Oxford, UK] and sourced from IDT (Integrated DNA Technologies, Leuven, Belgium) (Supplementary Material 1, Sheet: Primer_Seq_S2). During PCR, if multiple targets are amplified simultaneously in one pool and one AMR target is enriched in another, this might create a significant difference in molarity per amplicon during sequencing. Therefore, to avoid significant amplicon bias, up to seven AMR targets were incorporated in each pool, ensuring that no more than three targets are amplified at once in any given pool during PCR (Supplementary Material 2). This was achieved by combining primers for gene/variants of genes that are unlikely/less likely to be present in a bacterium at the same time.

### MultiSeq-AMR workflow tested on reference bacterial/fungal isolates

A total of 16 bacterial and 5 fungal reference/ATCC/NCTC strains with available whole-genome sequencing (WGS) data were chosen for initial benchmarking of AMR primer pools 1–7 and the species-specific pool (Fig. 1 and Supplementary Material 1, Sheet: Sample_info_S1). Pure bacterial/fungal isolates were initially grown overnight in Brain Heart Infusion and Sabouraud dextrose media (Sigma-Aldrich, MO, USA). In total, 1 ml overnight cultures were then centrifuged at 10 000***g*** for 5 min, and the pellets were resuspended in 400 µl DNA/RNA Shield (Zymo Research, CA, USA). DNA extraction was performed using QIAamp UCP Pathogen Mini kit with Pathogen Lysis Tubes L (Qiagen, Hilden, Germany) following the manufacturer’s recommendation. Two sterile human blood and one sterile H_2_O sample were also processed as negative controls alongside the 21 bacterial/fungal species. In total, 1 µl extracted DNA was quantified using the Qubit dsDNA broad range assay kit (Thermo Fisher Scientific, MA, USA) and 10 ng DNA was used as input/pool for PCR. Details for rapid PCR amplification/barcoding, library preparation and sequencing can be found in Supplementary Material 2.

### Validation of MultiSeq-AMR with BACT/ALERT positive samples

To further validate MultiSeq-AMR, 33 BACT/ALERT (bioMérieux, Marcy-l'Étoile, France) flagged positive blood culture and three negative blood culture media were processed as negative controls. All the culture-positive samples had sequencing results available through a previous study for comparison with MultiSeq-AMR [26]. Briefly, 400 µl enriched culture media samples were used directly for total DNA extraction using QIAamp UCP Pathogen Mini kit with Pathogen Lysis Tubes L (Qiagen, Hilden, Germany) according to manufacturer instructions. In total, 2 μl extracted DNA was used per PCR primer pool for amplification and barcoding. More details on cycling conditions, library preparation and sequencing can be found in Supplementary Material 2.

### Rapid enrichment combined with MultiSeq-AMR

To accelerate reporting time, a rapid culture enrichment method was tested using eight Gram-negative and seven Gram-positive ATCC/clinical isolates (Supplementary Material 1, Sheet: Rapid_enrichment_S3). For this, between 1 and 10 colony forming units (c.f.u.) of log phase cells were spiked in BACT/ALERT culture bottles pre-supplemented with 10 ml sterile sheep blood. Culture bottles were incubated and taken out at every 2 h interval for plating and colony counts for up to 10 h using the method described by Miles *et al.* [27]. All the culture experiments were done in triplicates, and during every sampling session, 1 ml enriched samples were aspirated from the culture bottles and preserved at −80 °C for further processing.

Finally, 13 ATCC/clinical isolates (seven Gram-positive and six Gram-negative) enriched for 6 h and preserved at −80 °C were chosen to be processed further with MultiSeq-AMR (Supplementary Material 1, Sheet: Sample_info_S1). To remove unwanted host DNA that might oversaturate the PCR reaction and to concentrate bacteria in the sample, a rapid host depletion step was performed for the 6 h enriched spiked blood (rapid enrichment blood culture) samples prior to DNA extraction (Supplementary Material 2). DNA extraction, PCR amplification and barcoding and library preparation were performed as described in Supplementary Material 2.

### Bioinformatic analysis to identify species and AMR determinants

To classify bacterial/fungal species and AMR determinants, fastq reads with ≥Q9 scores were initially trimmed to remove adapters using porechop (version 0.2.4) [28]. Adapter-trimmed fastq files were then processed with Kraken 2 [29] using the ‘EPI2ME wf-16S’ workflow’s 16S rRNA, 18S rRNA, 28S rRNA and internal transcribed spacer database to differentiate AMR and species-specific reads. Sequencing reads that matched with the Kraken 2 database were grouped as species, and unclassified reads at this stage were pooled separately as potential AMR determinants. Next, to overcome possible misidentification issues due to sequencing errors, which are relatively higher with ONT R9.4.1 kit chemistries, classified fastq reads were assembled *de novo* using Megahit (version 1.2.9) [30] with default parameters, and contigs smaller than 200 bp and having a multiplicity/depth less than 20 were dropped. Next, the filtered contigs were subjected to blastn (version 2.15.0) [31] using the NCBI nr database and information such as Subject Taxonomy IDs (staxids), Subject Scientific Name (ssciname), Subject Common Names (scomnames), Subject blast Names (sblastnames), Subject Super Kingdoms (sskingdoms), Subject Sequence ID (sseqid), Bit Score (bitscore), Alignment Length (length), Expectation Value (evalue) and Percentage Identity (pident) from the top 10 hits per contig were saved for further analysis (Supplementary Material 3). Finally, the most common ssciname/sscinames (representing ≥25% of all hits) per sample or barcode identified with blastn were reported as the causative bacterial/fungal species (Figs 1 and S1 in Supplementary Material 4).

Unclassified reads were directly analysed using ResFinder (version 4.5.0) with the ‘fastq (Nanopore Reads)’ option (‘--nanopore’) to identify AMR determinants. The identity and length (-*t* and -*l*) thresholds were lowered and set to 40% to allow for identification of MultiSeq-AMR amplicons that are ≤50% of the reference gene sequences present in the ResFinder database and might be missed during the search (Fig. 1). Classified AMR reads were finally compared to the sequencing (WGS) results obtained from our earlier study [26] or the genomes of the ATCC/reference strains. The accuracy of MultiSeq-AMR in identifying AMR determinants was assessed using four categories: false negative, false positive, true negative and true positive. Where false negative means that AMR genes were targeted by the MultiSeq-AMR panel and were present in the bacteria but were not identified, false positive means that genes/targets that are absent in the reference strain but detected with MultiSeq-AMR, true negative means that genes/targets that are not present and not supposed to be detected and remain undetected and true positive means that genes/targets that are present in both the panel and the bacteria and were identified with MultiSeq-AMR.

Instead of using sequencing time, which can vary depending on the type of sequencing flow cells used, yield in Mbp was used as a more consistent proxy. To understand the minimum sequencing yield in Mbp required (per sample) to predict species/AMR with the same/similar accuracy, all the sequencing reads were subsampled randomly (10, 8, 6, 4 and 2 Mbp) with rasusa (version 2.0.0) [32] and processed similarly.

### Statistical analysis

Statistical analysis was performed, and graphs were generated using R base (version 4.3.3) in RStudio (version 2023.12.1). R packages readr, readxl, dplyr, ggplot2, stringr, reshape2 and viridis were used for this purpose.

## Results

### MultiSeq-AMR identifies pathogens and AMR determinants with high accuracy

**Fig. 2.**
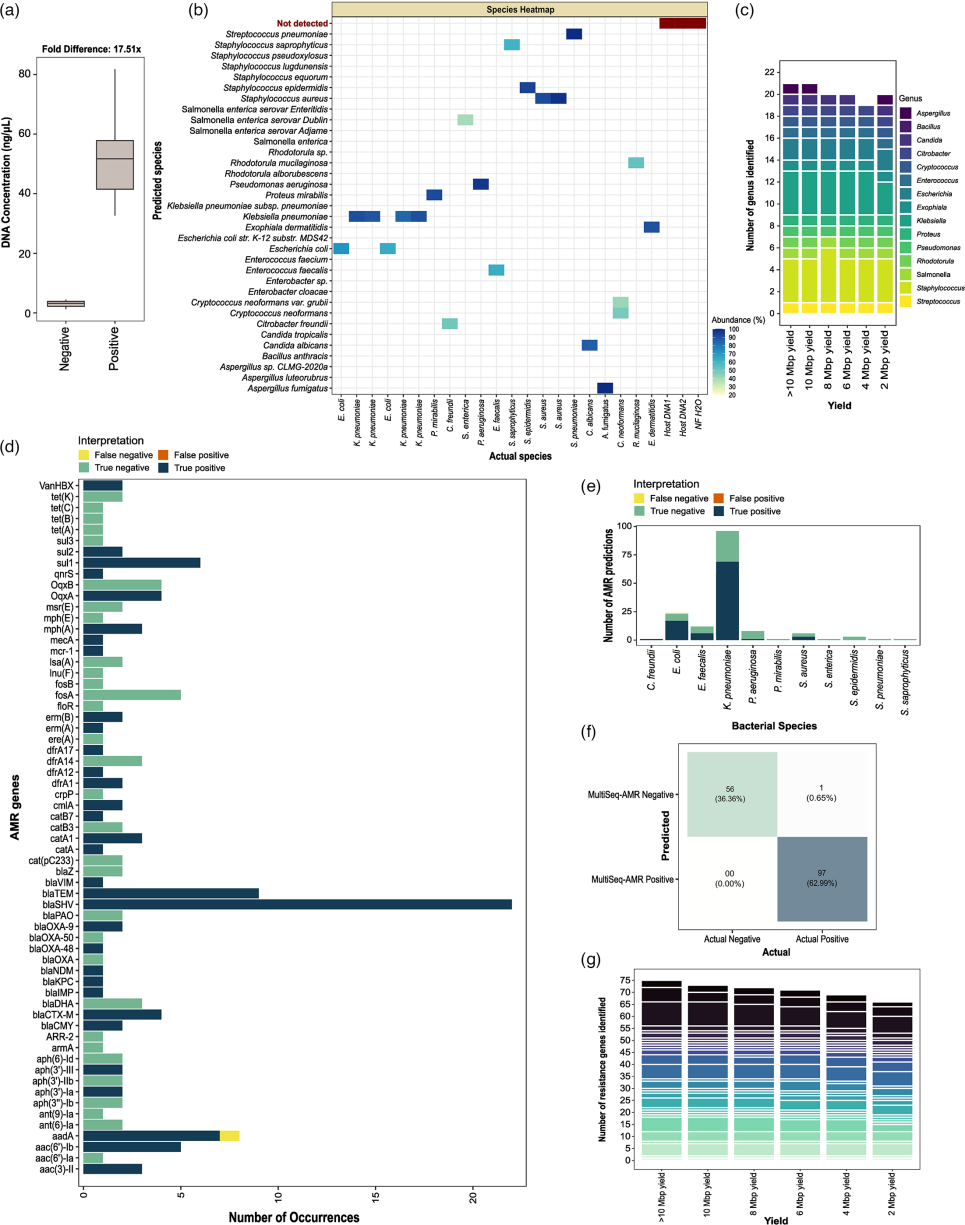
MultiSeq-AMR workflow tested on purified DNA from pure bacterial/fungal isolates. (**a**) DNA concentration of the bead-cleaned pooled PCR products, following amplification and barcoding, was measured for 21 bacterial/fungal isolates and negative controls (host DNA and H_2_O). (**b**) Bacterial/fungal species identified with MultiSeq-AMR (*y*-axis) versus known identity (ATCC stains) of species used for initial testing. The heatmap shows the proportion of blast hits matched to the species (those remaining after ≥25% abundance cutoff used in this study). (**c**) Number of genera predicted with different sequencing yields (>10, 10, 8, 6, 4 and 2 Mbp) using the same abundance cutoff. Bar plots are coloured by the type of bacterial/fungal genus identified with different sequencing yields. (**d)–(f**) Accuracy of MultiSeq-AMR in identifying AMR genes, determined by examining four categories: false negatives (yellow: AMR primers/genes present in both MultiSeq_AMR panel and the bacteria but undetected), false positives (orange: genes/targets that are absent in the reference bacteria but detected with MultiSeq_AMR), true negatives (green: genes that should not be detected with MultiSeq-AMR and remained undetected) and true positives (deep blue: targets/genes that are present in both the panel and the bacteria and identified with MultiSeq_AMR). The same categories were compared across (d) antibiotic genes, (e) bacterial species and (f) combined to see overall performance. (**g**) Number of AMR genes identified in samples with varying sequencing yield (>10, 10, 8, 6, 4 and 2 Mbp).

Barcoded and bead-cleaned PCR pools prepared from genomic DNA of pure bacterial/fungal isolates had 17.5-fold higher DNA concentration (positive: 51.93 ng µl^−1^ versus control: 2.96 ng µl^−1^) on average compared to human DNA and negative controls (Fig. 2a). All the bacterial (16/16) and fungal (5/5) isolates tested were identified accurately at both genus and species levels. Both host DNA and H_2_O negative controls did not yield contigs after assembly and quality filtering and therefore were identified as negative (3/3). A median of 86.66% (sd±22.24%) blast contig hits (ssciname) per sample was assigned to the correct species used for benchmarking (Fig. 2b). With 10 Mbp sequencing yield per sample, MultiSeq-AMR identified all 21 positive and 3 negative samples accurately. Even with only 2 Mbp yield, 95% (20/21) bacteria were accurately determined (Fig. 2c).

For AMR gene identification, MultiSeq-AMR showed 99.4% categorical agreement (CA) (153/154 predictions) overall when compared to the genes present in the reference strains. Of 98 AMR genes present among the reference strains that we expected should be predicted by MultiSeq-AMR, only 1 was not identified. This single false negative occurred for AMR gene *aadA* (1/8 occurrences), present in one of the *Escherichia coli* strains benchmarked here (Fig. 2d–f). With 10, 8, 6, 4 and 2 Mbp yield, MultiSeq-AMR identified AMR genes with 96.05, 94.73, 93.42, 90.78 and 86.84% sensitivity, respectively, similar to the results obtained with greater (20–50 Mbp) sequencing yield (Fig. 2g).

### Species and AMR genes can be accurately identified with BACT/ALERT positive samples

**Fig. 3.**
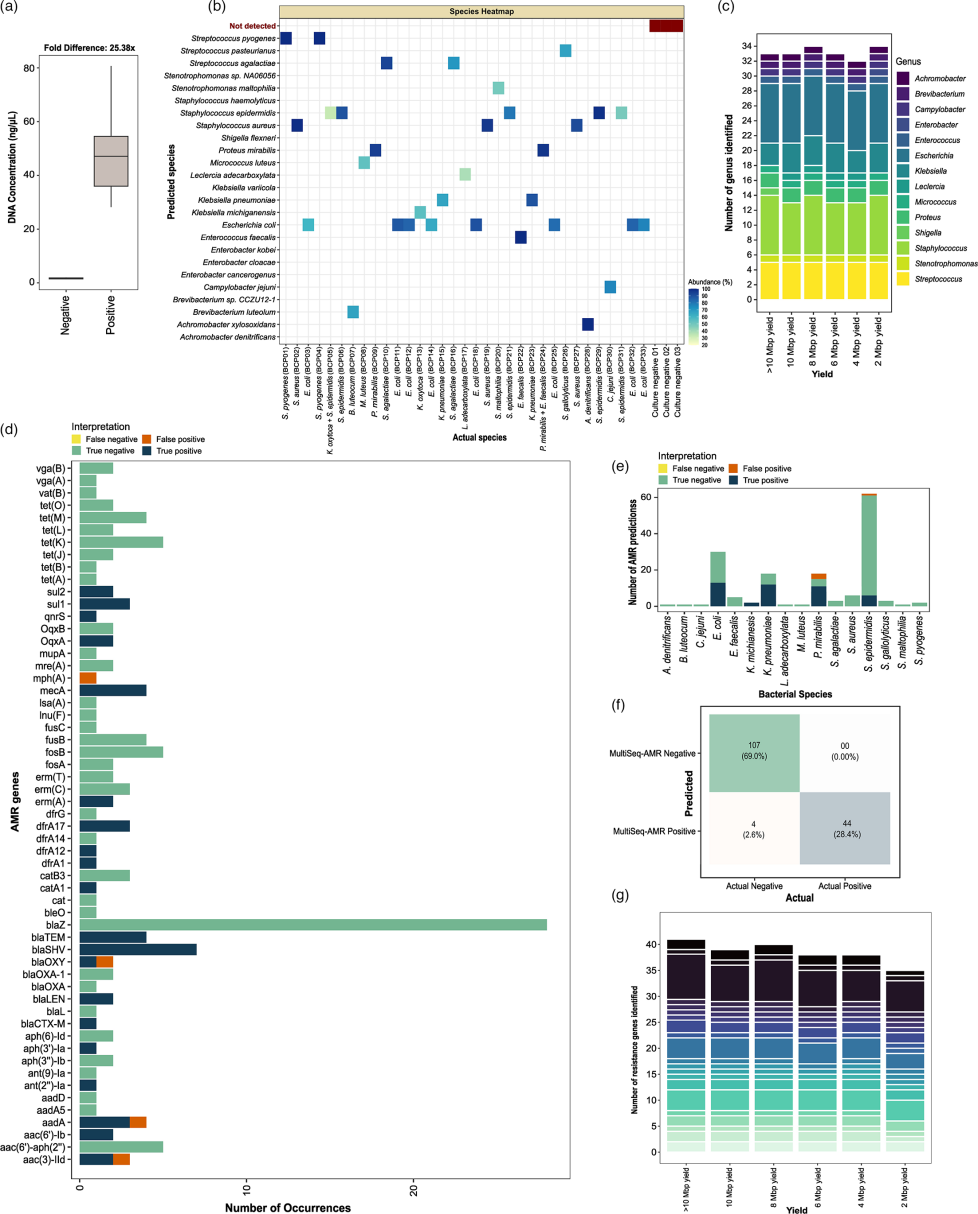
MultiSeq-AMR workflow tested on BACT/ALERT positive and negative samples. (**a**) DNA concentration of pooled PCR products, after PCR amplification and barcoding, was determined for 33 BACT/ALERT positive and 3 negative samples. (**b**) Pathogens identified with MultiSeq-AMR (*y*-axis) versus standard blood culture-based results (*x*-axis) utilized for initial reporting. The heatmap shows the proportion of blast hits matched to the species (those remaining after ≥25% abundance cutoff). (**c**) Number of bacterial genera predicted with different sequencing yields (>10, 10, 8, 6, 4 and 2 Mbp) using the same threshold. Bar plots are coloured by the type of bacterial genus identified with >10–2 Mbp sequencing yield. (**d)–(f**) Accuracy of MultiSeq-AMR in identifying AMR genes determined by examining four categories: false negatives (yellow: AMR primers/genes present in both MultiSeq_AMR panel and the bacteria but undetected), false positives (orange: genes/targets that are absent in the reference bacteria but detected with MultiSeq_AMR), true negatives (green: genes that are supposed to be undetected and not identified with MultiSeq-AMR) and true positives (deep blue: targets/genes that are present in both the panel and the bacteria and identified with MultiSeq_AMR). The same categories were compared across (d) antibiotic genes, (e) bacterial species and (f) combined to see overall performance. (**g**) Number of AMR genes identified in samples with varying sequencing yield (>10, 10, 8, 6, 4 and 2 Mbp).

With BACT/ALERT culture media samples (*n*=36), PCR amplification led to 25.4-fold difference in DNA concentration between flagged positive (*n*=33) and negative (*n*=3) samples tested (culture positives: 47.99 ng µl^−1^ versus culture negatives: 1.69 ng µl^−1^) (Fig. 3a). For mono-bacterial samples, 100% (31/31) genus and 96.77% (30/31) species were accurately identified with MultiSeq-AMR. Overall, 2/4 genera and species were accurately detected from two mixed infection samples; the remaining two species identified by bacteriological culture, i.e. *Klebsiella oxytoca* and *Enterococcus faecalis*, were not identified with our protocol. All three negative samples were called negative, as no contigs were retained following assembly and quality filtering. A median of 81.51% (sd±21.87%) blast contig hits matched the species identified with culture (Fig. 3b). With only 2 Mbp reads, bacterial prediction accuracy remained similar (>95%) to the results obtained from 20 to 50 Mbp sequencing yield per sample (Fig. 3c).

MultiSeq-AMR exhibited 97.4% CA (151/155 predictions) for predicting AMR genes in the 33 BACT/ALERT positive samples. Unlike for the reference isolates tested, no false negative predictions were observed. However, resistance genes were inaccurately identified in four instances, leading to an 8% (4/48) false positivity rate. Genes *mph(A*) (1/1), *blaOXY* (1/2), *aadA* (1/4) and *aac(3)-IId* (1/3) were falsely identified in two samples positive for *Proteus mirabilis* and *Staphylococcus epidermidis* (Fig. 3d–f). In total, ~95% AMR genes (sd±2.2%) identified with 20–50 Mbp sequencing yield were still detected with 4–10 Mbp and 85.36% with only 2 Mbp sequencing yield per sample (Fig. 3g).

### MultiSeq-AMR with rapid culture enrichment can facilitate same-day microbiological diagnosis

With spiked sheep blood as host matrix, generation time for both Gram-positive (*n*=7) and Gram-negative (*n*=8) organisms tested for rapid culture enrichment was 22.86 (range 14.46–32.88 min) and 20.48 (range 16.26–26.04 min) min on average, respectively. In total, 4 h BACTEC enrichment led to a median concentration of 1.2×10^1^ c.f.u. ml^−1^ for Gram-positive and 1.2×10^2^ c.f.u. ml^−1^ in case of Gram-negative bacterial species. With 6 h culture, Gram-positive organisms reached a median of 7.0×10^2^ (from 1.15×10^2^ to 3.02×10^4^) c.f.u. ml^−1^ and negatives 3.4×10^3^ (between 5.83×10^1^ and 2.11×10^4^) c.f.u. ml^−1^. Of all the species tested, *Pseudomonas aeruginosa* exhibited the slowest growth, reaching only 5.83×10^1^ c.f.u. ml^−1^ with 6 h enrichment. Median bacterial concentration reached 2.65×10^6^ c.f.u. ml^−1^ (between 7.50×10^4^ and 9.27×10^9^) for Gram-positive and 9.56×10^6^ c.f.u. ml^−1^ (from 4.33×10^5^ to 1.50×10^9^) for Gram-negative organisms with 10 h BACTEC enrichment (Supplementary Material 1, Sheet: Rapid_enrichment_S3).

**Fig. 4.**
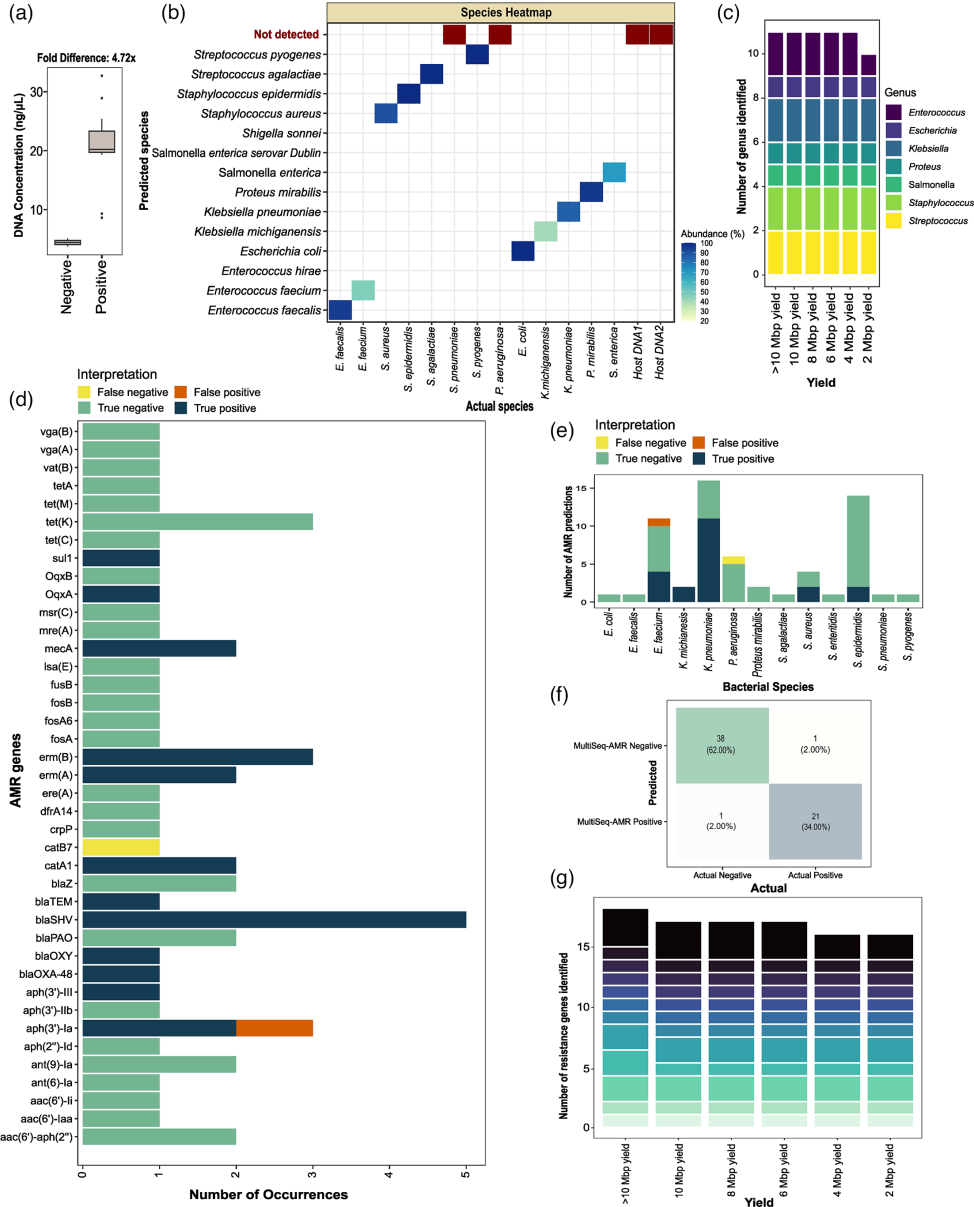
MultiSeq-AMR workflow tested on rapid culture-enriched (6 h) spiked blood samples. (**a**) DNA concentration of pooled PCR products, after PCR amplification and barcoding, was determined for 7 Gram-positive, 6 Gram-negative bacterial species and 2 sterile rapid enriched whole blood samples. (**b**) Bacterial species identified with MultiSeq-AMR (*y*-axis) versus species initially used for spiking and rapid enrichment (*x*-axis). The heatmap shows the proportion of blast hits matched to the species remaining after ≥25% abundance threshold. (**c**) Number of genera predicted with different sequencing yields (>10, 10, 8, 6, 4 and 2 Mbp) using the same threshold. Bar plots are coloured by the type of bacterial genus identified with varying sequencing yield (>10–2 Mbp). (**d)–(f**) Accuracy of MultiSeq-AMR in identifying AMR genes determined by examining four categories: false negatives (yellow: AMR primers/genes present in both MultiSeq_AMR panel and the bacteria but unidentified), false positives (orange: genes/targets that are absent in the reference bacteria but detected with MultiSeq_AMR), true negatives (green: genes that are not supposed to be detected by the panel and remained undetected) and true positives (deep blue: targets/genes that are present in both the panel and the bacteria and identified with MultiSeq_AMR). The same categories were compared across (**d**) bacterial species, (**e**) antibiotic genes and (**f**) combined to see overall performance. (**g**) Number of AMR genes identified in samples with different sequencing yields (>10, 10, 8, 6, 4 and 2 Mbp).

With 6 h rapid enrichment, DNA concentration between positive (spiked samples; *n*=13) and negative (sterile enriched sheep blood; *n*=2) samples differed 4.7-fold (positives: 20.76 ng µl^−1^ and negatives: 4.4 ng µl^−1^) on average after PCR amplification with MultiSeq-AMR primers. Two samples, spiked with *P. aeruginosa* and *Streptococcus pneumoniae*, had significantly lower DNA concentration (average 8.95 ng µl^−1^) after PCR compared to other bacterial species (average 23.05 ng µl^−1^) (Fig. 4a). MultiSeq-AMR combined with rapid culture enrichment identified 84.61% (11/13) bacteria accurately at both genus/species levels, with a median 90.0% (sd±24.77%) blastn hits assigned to the species used for spiking. *P. aeruginosa* and *S. pneumoniae*, the two species yielding suboptimal amplicons/DNA concentration during PCR, were not detected, as assembled contigs did not pass the initial quality threshold for blastn. The two negative controls did not flag positive, as expected (Fig. 4b). The accuracy of bacterial species identification was the same with as low as 4 Mbp yield as obtained with 20–50 Mbp yield per sample (84.61%; 11/13 species). With 2 Mbp sequencing yield, MultiSeq-AMR detected 10/13 bacteria accurately. *Enterococcus faecium*, in addition to *P. aeruginosa* and *S. pneumoniae*, was missed (Fig. 4c).

Overall, 96% CA (59/61 predictions) was observed for AMR gene identification. One false negative (1/39) and one false positive (1/22) prediction were obtained from genes *catB7* and *aph(3′)-Ia*, respectively. *E. faecium and P. aeruginosa* were two of the bacterial species exhibiting false positive and false negative gene predictions (Fig. 4d–f). With 10, 8 and 6 Mbp yield, 94.1% (16/17) genes matched with the 20–50 Mbp yield. Up to 88.2% concordance was seen with only 4 and 2 Mbp sequencing yield (Fig. 4g).

## Discussion

BSIs are among the leading causes of mortality and morbidity worldwide and require timely microbiological diagnosis for more precise and effective antibiotic treatment leading to better patient outcomes. In working towards that aim, this study introduced MultiSeq-AMR, a rapid nanopore amplicon-sequencing workflow to detect bacterial and fungal species and a comprehensive set of AMR determinants to aid and accelerate existing methods for diagnosing BSI. MultiSeq-AMR leverages the rapid, high throughput and real-time sequencing capability of ONT platforms. The use of rapid PCR barcoding and library preparation steps enables sample preparation in ~4 h, with sequence data available for analysis and possible result reporting almost immediately after sequencing begins. The choice of oligonucleotides includes 2 universal bacterial (16S rRNA) and fungal (28S rRNA) and 91 clinically important AMR gene targets, covering 11 antibiotic classes. We validated MultiSeq-AMR using reference fungal and bacterial strains, BACT/ALERT positive and negative clinical samples from individuals with suspected BSI and rapid culture-enriched spiked whole blood samples.

There are various bioinformatic options available for identifying bacterial and fungal species utilizing 16S/28S rRNA reference datasets [29,33]. However, only a few have been standardized for BSI diagnosis and/or tailored to work on mixed amplicon-sequencing reads. Therefore, benchmarking these workflows was not within the scope of this study. Instead, we utilized a simple bioinformatic approach that utilizes some of the well-reputed tools to identify species and AMR determinants to evaluate the utility of MultiSeq-AMR. Later, this workflow/cutoff can be modified or replaced with newer alternatives if required. Of all the possible cutoffs, ≥25% abundance threshold of blast contig hits showed the highest sensitivity and specificity for genus and species detection (Fig. S1 in Supplementary Material 4). With this threshold, MultiSeq-AMR detected 21 bacterial/fungal reference strains and 31 mono-bacterial BSI samples with a genus and species level accuracy of 100 and 98.4%. Even with as little as 2 Mbp sequencing yield per sample, genus-level prediction was ≥95%. For two BACT/ALERT mixed species samples (BCP05 and BCP24) having two bacterial species each, only one was detected. This is likely due to the low abundance of the two species that were missed (*K. oxytoca* and *E. faecalis*) compared to the other two (*S. epidermidis* and *P. mirabilis*) identified with MultiSeq-AMR. With a reduced abundance threshold, we were able to recover both *K. oxytoca* and *S. epidermidis* from sample BCP05; however, this adjustment also resulted in reduced specificity in other samples (data not shown). As a result, it was decided to keep the existing cutoff (≥25%) to achieve the best overall specificity. For the rapid (6 h) culture-enriched spiked blood, species from 11 out of 13 positive samples were correctly identified (sensitivity: 84.61%). No bacteria were detected from two samples spiked with *P. aeruginosa* and *S. pneumoniae*, exhibiting false negativity. This may be a result of the slow growth rate of some of the species/strains used for the rapid enrichment experiment. For example, *P. aeruginosa* reached 5.83×10¹ c.f.u. ml^−1^ after 6 h of BACTEC enrichment. This concentration is possibly too low for the DNA extraction and PCR kit to reliably extract/amplify target regions. These two species also had significantly lower DNA concentration after PCR barcoding (8.95 versus 23.05 ng µl^−1^ for other samples). This means the 6 h enrichment method may not sufficiently enrich slow-growing bacterial/fungal species [34] and therefore be falsely identified as negative. However, because MultiSeq-AMR is aimed to be used alongside traditional methods, it is likely that these missed organisms would be identified later with culture. It is also worth noting that there might also be many bacterial species not tested with the rapid enrichment method that could potentially reach the desired threshold for robust capture and detection with MultiSeq-AMR. A large collection of clinical samples with multiple strains/genotypes from the same species would ideally be used to more fully validate rapid enrichment with MultiSeq-AMR in the future, as spiked sheep blood may not fully represent freshly drawn blood samples from suspected BSI individuals and/or accurately reflect bacterial growth rate.

Similarly to the approaches described for species identification, there are multiple methods available for detecting AMR genes from sequencing reads [35,37]. Again, rather than assess multiple tools, we implemented a well-reputed tool/database, ResFinder, to preliminarily test the utility of MultiSeq-AMR. While comprehensive benchmarking could help identify which bioinformatic tool has the best overall performance, this was outside the scope of the present study. By default, ResFinder 4.0 has a ‘Threshold for %ID’ of 90% and a 60% ‘Minimum length’ cutoff. Because MultiSeq-AMR generates smaller amplicons that could fall well below the default threshold for some targets (e.g. *mecA* and *mecC*), we gradually reduced these values to determine those that achieved the greatest accuracy, which resulted in the selection of 40% ‘Threshold for %ID’ and ‘Minimum length’ (Fig. S2 in Supplementary Material 4). With this selected cutoff, MultiSeq-AMR achieved 98.4% CA overall for extracted genomic DNA and BACT/ALERT positive samples across 49 AMR targets grouped into 7 primer pools. Even with 6 h rapid culture enrichment, 59/61 expected AMR genes were correctly identified, with only one false negative and one false positive prediction. The false negative AMR results observed in this study may be attributed to primer efficiency, as no AMR hits from those genes were found in the raw sequence datasets. False positivity could stem from cross-target non-specific amplification or contamination, highlighting the importance of stringent laboratory practices. Up to 85% of genes were identified with only 2 Mbp sequencing yield for all the sample types, meaning the sequencing time to generate AMR and species reports could be almost instantaneous following a sequencing run. That being said, a large number of clinical samples need to be tested, especially for AMR determinants, to draw reliable conclusions regarding the performance of MultiSeq-AMR.

A somewhat costly disadvantage of the rapid enrichment method could be the requirement to sequence all enriched blood samples from suspected BSI patients blindly until sufficient sequencing reads are generated for analysis in order to determine sample positivity or negativity (since these would be tested before culture bottles flag positive). However, when we compared positive (containing pathogen) and negative samples (culture-negative or sterile blood sample), the DNA concentration after PCR barcoding and cleanup was at least 5 to 20 times higher in samples containing detectable DNA quantities of a given pathogen. This provides a unique signature, which could potentially be exploited to select and sequence only the samples with significantly higher post-PCR DNA concentration compared to the negative controls to save costs. However, this approach must be validated/calibrated site-wise using more patient samples, including both positive and negative cases, across different clinical contexts before implementation.

It is important to acknowledge a few limitations of this study. Of the 13 AMR pools designed as part of this study, we successfully benchmarked 7 (*n*=49 targets) using reference fungal and bacterial strains, BACT/ALERT positive and negative samples and rapid (6 h) culture-enriched spiked samples. The remaining 6 AMR pools covering 42 gene targets could not be validated due to the lack of sufficient positive controls. In the future, the validation of these additional pools (8–13) with appropriate positive and negative controls would greatly expand the AMR gene panel that could be tested with this approach. Because all the primers/pools were designed similarly, it is likely that most primer sets would amplify successfully, but primer failure or reduced amplification efficiency cannot be ruled out. Also, the MultiSeq-AMR workflow was validated using R9.4.1 flow cells and supported sequencing kits, whereas more accurate R10.4.1 kits became available halfway through the study. We believe MultiSeq-AMR can easily be adapted for R10.4.1 flow cells and kit chemistries, and the overall performance would benefit from more accurate (≥Q20) sequencing reads for predicting species and AMR determinants, although this must be validated and confirmed.

Taken together, this study demonstrated that MultiSeq-AMR is highly effective in detecting bacterial and fungal species and clinically important antibiotic-resistant genes from samples such as extracted genomic DNA, BACT/ALERT flagged positive culture media and rapid culture-enriched samples for a large group of clinically relevant pathogens. With only ~4 h of hands-on time, MultiSeq-AMR can enable microbiological diagnosis under 5 h of BACT/ALERT positivity or 11 h of sample collection, given the availability of standardized real-time bioinformatics workflows. The choice of AMR targets can be tailored and extended indefinitely, pathogen/syndrome-wise, and hands-on time could be further reduced through automation, making this approach highly modular. Consequently, as a rapid, cost-effective and modular workflow, MultiSeq-AMR has potential in improving outcomes for critically ill patients with BSI.

## Supplementary material

10.1099/mgen.0.001383 Supplementary Material 3.

10.1099/mgen.0.001383 Supplementary Material 4.

10.1099/mgen.0.001383 Supplementary Material 1.

10.1099/mgen.0.001383 Supplementary Material 2.

## Abbreviations

AMR: antimicrobial resistance
ATCC: American Type Culture Collection
BSI: bloodstream infection
CA: categorical agreement
c.f.u.: colony forming units
mNGS: metagenomic next-generation sequencing
NCBI: National Center for Biotechnology Information
NGS: next-generation sequencing
ONT: Oxford Nanopore Technologies
WGS: whole-genome sequencing

## References

[1] Diekema DJ, Hsueh P-R, Mendes RE, Pfaller MA, Rolston KV (2019). The microbiology of bloodstream infection: 20-year trends from the SENTRY antimicrobial surveillance program. Antimicrob Agents Chemother.

[2] Ikuta KS, Swetschinski LR, Robles Aguilar G, Sharara F, Mestrovic T (2022). Global mortality associated with 33 bacterial pathogens in 2019: a systematic analysis for the global burden of disease study 2019. The Lancet.

[3] Sakr Y, Jaschinski U, Wittebole X, Szakmany T, Lipman J (2018). Sepsis in intensive care unit patients: worldwide data from the intensive care over nations audit. Open Forum Infect Dis.

[4] Scheer CS, Fuchs C, Gründling M, Vollmer M, Bast J (2019). Impact of antibiotic administration on blood culture positivity at the beginning of sepsis: a prospective clinical cohort study. Clin Microbiol Infect.

[5] Strich JR, Heil EL, Masur H (2020). Considerations for empiric antimicrobial therapy in sepsis and septic shock in an era of antimicrobial resistance. J Infect Dis.

[6] Ohnuma T, Chihara S, Costin B, Treggiari MM, Bartz RR (2023). Association of appropriate empirical antimicrobial therapy with in-hospital mortality in patients with bloodstream infections in the US. JAMA Netw Open.

[7] Salam MA, Al-Amin MY, Salam MT, Pawar JS, Akhter N (2023). Antimicrobial resistance: a growing serious threat for global public health. Healthcare.

[8] Paharik AE, Schreiber Hlt, Spaulding CN, Dodson KW, Hultgren SJ (2017). Narrowing the spectrum: the new frontier of precision antimicrobials. Genome Med.

[9] D’Andreano S, Cuscó A, Francino O (2021). Rapid and real-time identification of fungi up to species level with long amplicon nanopore sequencing from clinical samples. Biol Methods Protoc.

[10] Ali J, Johansen W, Ahmad R (2024). Short turnaround time of seven to nine hours from sample collection until informed decision for sepsis treatment using nanopore sequencing. Sci Rep.

[11] Bauer MJ, Peri AM, Lüftinger L, Beisken S, Bergh H (2022). Optimized method for bacterial nucleic acid extraction from positive blood culture broth for whole-genome sequencing, resistance phenotype prediction, and downstream molecular applications. J Clin Microbiol.

[12] Sajib MSI, Brunker K, Oravcova K, Everest P, Murphy ME (2024). Advances in host depletion and pathogen enrichment methods for rapid sequencing-based diagnosis of bloodstream infection. J Mol Diagn.

[13] Zhang Y, Lu X, Tang LV, Xia L, Hu Y (2023). Nanopore-targeted sequencing improves the diagnosis and treatment of patients with serious infections. mBio.

[14] Zhao K, Tu C, Chen W, Liang H, Zhang W (2022). Rapid identification of drug-resistant tuberculosis genes using direct PCR amplification and Oxford Nanopore Technology sequencing. Can J Infect Dis Med Microbiol.

[15] Zhang C, Xiu L, Li Y, Sun L, Li Y (2021). Multiplex PCR and nanopore sequencing of genes associated with antimicrobial resistance in *Neisseria gonorrhoeae* directly from clinical samples. Clin Chem.

[16] Smith SD, Choi J, Ricker N, Yang F, Hinsa-Leasure S (2022). Diversity of antibiotic resistance genes and transfer elements-quantitative monitoring (DARTE-QM): a method for detection of antimicrobial resistance in environmental samples. Commun Biol.

[17] Li Y, Shi X, Zuo Y, Li T, Liu L (2022). Multiplexed target enrichment enables efficient and in-depth analysis of antimicrobial resistome in metagenomes. Microbiol Spectr.

[18] Callahan BJ, Wong J, Heiner C, Oh S, Theriot CM (2019). High-throughput amplicon sequencing of the full-length 16S rRNA gene with single-nucleotide resolution. Nucleic Acids Res.

[19] Romanelli AM, Fu J, Herrera ML, Wickes BL (2014). A universal DNA extraction and PCR amplification method for fungal rDNA sequence-based identification. Mycoses.

[20] Edgar RC (2004). MUSCLE: multiple sequence alignment with high accuracy and high throughput. Nucleic Acids Res.

[21] Ye J, Coulouris G, Zaretskaya I, Cutcutache I, Rozen S (2012). Primer-BLAST: a tool to design target-specific primers for polymerase chain reaction. BMC Bioinformatics.

[22] Boyd DA, Tyler S, Christianson S, McGeer A, Muller MP (2004). Complete nucleotide sequence of a 92-kilobase plasmid harboring the CTX-M-15 extended-spectrum beta-lactamase involved in an outbreak in long-term-care facilities in Toronto, Canada. Antimicrob Agents Chemother.

[23] Jensen LB, Frimodt-Møller N, Aarestrup FM (1999). Presence of erm gene classes in gram-positive bacteria of animal and human origin in Denmark. FEMS Microbiol Lett.

[24] Bansal S, Tandon V (2011). Contribution of mutations in DNA gyrase and topoisomerase IV genes to ciprofloxacin resistance in *Escherichia coli* clinical isolates. Int J Antimicrob Agents.

[25] Ng LK, Martin I, Alfa M, Mulvey M (2001). Multiplex PCR for the detection of tetracycline resistant genes. Mol Cell Probes.

[26] Sajib MSI, Oravcova K, Brunker K, Everest P, Fuentes M (2009). Rapid and modular workflows for same-day sequencing-based detection of bloodstream infections and antimicrobial resistance determinants. medRxiv.

[27] Miles AA, Misra SS, Irwin JO (1938). The estimation of the bactericidal power of the blood. J Hyg.

[28] Wick RR, Judd LM, Gorrie CL, Holt KE (2017). Completing bacterial genome assemblies with multiplex MinION sequencing. Microb Genom.

[29] Wood DE, Lu J, Langmead B (2019). Improved metagenomic analysis with Kraken 2. Genome Biol.

[30] Li D, Liu CM, Luo R, Sadakane K, Lam TW (2015). MEGAHIT: an ultra-fast single-node solution for large and complex metagenomics assembly via succinct de Bruijn graph. Bioinformatics.

[31] Altschul SF, Gish W, Miller W, Myers EW, Lipman DJ (1990). Basic local alignment search tool. J Mol Biol.

[32] Hall M (2022). Rasusa: randomly subsample sequencing reads to a specified coverage. J Open Source Softw.

[33] Li H (2018). Minimap2: pairwise alignment for nucleotide sequences. Bioinformatics.

[34] Somily AM, Habib HA, Torchyan AA, Sayyed SB, Absar M (2018). Time-to-detection of bacteria and yeast with the BACTEC FX versus BacT/Alert Virtuo blood culture systems. Ann Saudi Med.

[35] Alcock BP, Huynh W, Chalil R, Smith KW, Raphenya AR (2023). CARD 2023: expanded curation, support for machine learning, and resistome prediction at the comprehensive antibiotic resistance database. Nucleic Acids Res.

[36] Bortolaia V, Kaas RS, Ruppe E, Roberts MC, Schwarz S (2020). ResFinder 4.0 for predictions of phenotypes from genotypes. J Antimicrob Chemother.

[37] Clausen PTLC, Aarestrup FM, Lund O (2018). Rapid and precise alignment of raw reads against redundant databases with KMA. BMC Bioinformatics.

