# Supplementary material for: MultiSeq-AMR: a modular amplicon-sequencing workflow for rapid detection of bloodstream infection and antimicrobial resistance markers
Source: Microb Genom. 2025 Apr 3;11(4):001383. doi: 10.1099/mgen.0.001383 (PMC12452178; doi:10.1099/mgen.0.001383)
Supplement: Supplementary Material 3. [file mgen-11-01383-s001.pdf]

Dependencies

Porechop: https://github.com/rrwick/Porechop
Kraken2: https://github.com/DerrickWood/kraken2
MEGAHIT: https://github.com/voutcn/megahit
BLASTn: https://github.com/OpenHero/gblastn
ResFinder: https://github.com/genomicepidemiology/resfinder
Others: AWK, pandas, openpyxl

Input and output directory setup

```
# Prompt for input/output directories and Kraken/BLAST database path
read -p "Enter input directory (FASTQ files): " input_dir
read -p "Enter output directory: " output_dir
read -p "Enter Kraken database path (16S, ITS, 18S and 28S database): " kraken_db
read -p "Enter BLAST database path (nr): " blast_db
read -p "Enter ResFinder database directory: " db_dir

# Create necessary directories
mkdir -p "$output_dir/trimmed_fastq" "$output_dir/kraken_output"
```

Adapter trimming using porechop

```
# Loop through all fastq files and trim adapters
for fastq_file in "$input_dir"/*.fastq.gz; do
  sample=$(basename "$fastq_file" .fastq.gz)
  trimmed_file="$output_dir/trimmed_fastq/${sample}_trimmed.fastq.gz"
  porechop -i "$fastq_file" -o "$trimmed_file"
  echo "Trimmed adapters for $sample"
done
```

Taxonomic classification using kraken2 (16S, ITS, 18S and 28S database)

```
# Classify reads with Kraken2
for trimmed_file in "$output_dir/trimmed_fastq"/*.fastq.gz; do
  sample=$(basename "$trimmed_file" _trimmed.fastq.gz)
  kraken_dir="$output_dir/kraken_output/$sample"
  mkdir -p "$kraken_dir"

  kraken2 --db "$kraken_db" \
    --output "$kraken_dir/${sample}_kraken_report.txt" \
    --report "$kraken_dir/${sample}_kraken_summary.txt" \
    --classified-out "$kraken_dir/${sample}_classified.fastq" \
    --unclassified-out "$kraken_dir/${sample}_unclassified.fastq" \
    --threads 8 --gzip-compressed "$trimmed_file"

  echo "Classified reads for $sample"
done
```

Assembly of classified reads

```
# Assemble classified reads with MEGAHIT
for classified_file in "$output_dir/kraken_output"/*/*_classified.fastq.gz; do
  sample=$(basename "$classified_file" _classified.fastq.gz)
  assembly_dir="$output_dir/assemblies/$sample"
```

```
    mkdir -p "$assembly_dir"
    megahit -r "$classified_file" -o "$assembly_dir"
    echo "Assembled reads for $sample"
done
```

Drop low quality reads and species Identification Using BLASTn

```
# BLASTn for species identification
for contigs_file in "$output_dir/assemblies"/*/final.contigs.fa; do
    sample=$(basename "$(dirname "$contigs_file")")
    blast_output="$output_dir/blast_results/${sample}_blast_results.txt"
    mkdir -p "$output_dir/blast_results"

    blastn -query "$contigs_file" -db "$blast_db" -out "$blast_output" \
        -outfmt '6 staxids sscinames scomnames sblastnames sskingdoms qseqid sseqid
bitscore length evalue pident' \
        -num_threads 8 -max_target_seqs 10
    echo "BLASTn completed for $sample"
done
```

Compile and filter BLAST results

```
# Compile BLAST results
combined_csv="$output_dir/blast_results/all_blast_results.csv"
echo
"sample_id,staxids,sscinames,scomnames,sblastnames,sskingdoms,qseqid,sseqid,bitscore,le
ngth,evalue,pident" > "$combined_csv"

for blast_result in "$output_dir/blast_results"/*_blast_results.txt; do
    sample=$(basename "$blast_result" _blast_results.txt)
    awk -v sample_id="$sample" 'BEGIN {FS=OFS="\t"} {print sample_id, $0}' "$blast_result" |
tr '\t' ',' >> "$combined_csv"
done

# Filter results using Python
python3 <<EOF
import pandas as pd
csv_file = "$combined_csv"
filtered_file = "$output_dir/blast_results/filtered_blast_results.csv"
df = pd.read_csv(csv_file)
df[df['pident'] >= 95].to_csv(filtered_file, index=False)
print(f"Filtered results saved to {filtered_file}")
EOF
```

AMR gene identification using resFinder

```
# AMR analysis using ResFinder
for unclassified_file in "$output_dir/kraken_output"/*/*_unclassified.fastq.gz; do
    sample=$(basename "$unclassified_file" _unclassified.fastq.gz)
    resfinder_dir="$output_dir/resfinder/$sample"
    mkdir -p "$resfinder_dir"

    python -m resfinder \
      -o "$resfinder_dir" -ifq "$unclassified_file" \
      --nanopore -l 0.6 -t 0.9 --acquired
    echo "ResFinder completed for $sample"
```

done

Compile resfinder results

```
# Aggregate ResFinder results
python3 <<EOF
import os
import pandas as pd
output_dir = "$output_dir/resfinder"
compiled_file = os.path.join(output_dir, "compiled_resfinder_results.xlsx")
all_data = []

for sample_dir in os.listdir(output_dir):
    sample_path = os.path.join(output_dir, sample_dir)
    if os.path.isdir(sample_path):
        pheno_file = os.path.join(sample_path, "pheno_table.txt")
        if os.path.exists(pheno_file) and os.stat(pheno_file).st_size > 0:
            df = pd.read_csv(pheno_file, sep='\t', skiprows=16)
            df['sample'] = sample_dir
            all_data.append(df)

if all_data:
    pd.concat(all_data).to_excel(compiled_file, index=False)
    print(f"ResFinder results compiled to {compiled_file}")
else:
    print("No phenotype results found.")
EOF
```
