# Supplementary material for: MultiSeq-AMR: a modular amplicon-sequencing workflow for rapid detection of bloodstream infection and antimicrobial resistance markers
Source: Microb Genom. 2025 Apr 3;11(4):001383. doi: 10.1099/mgen.0.001383 (PMC12452178; doi:10.1099/mgen.0.001383)
Supplement: Supplementary Material 4. [file mgen-11-01383-s002.pdf]

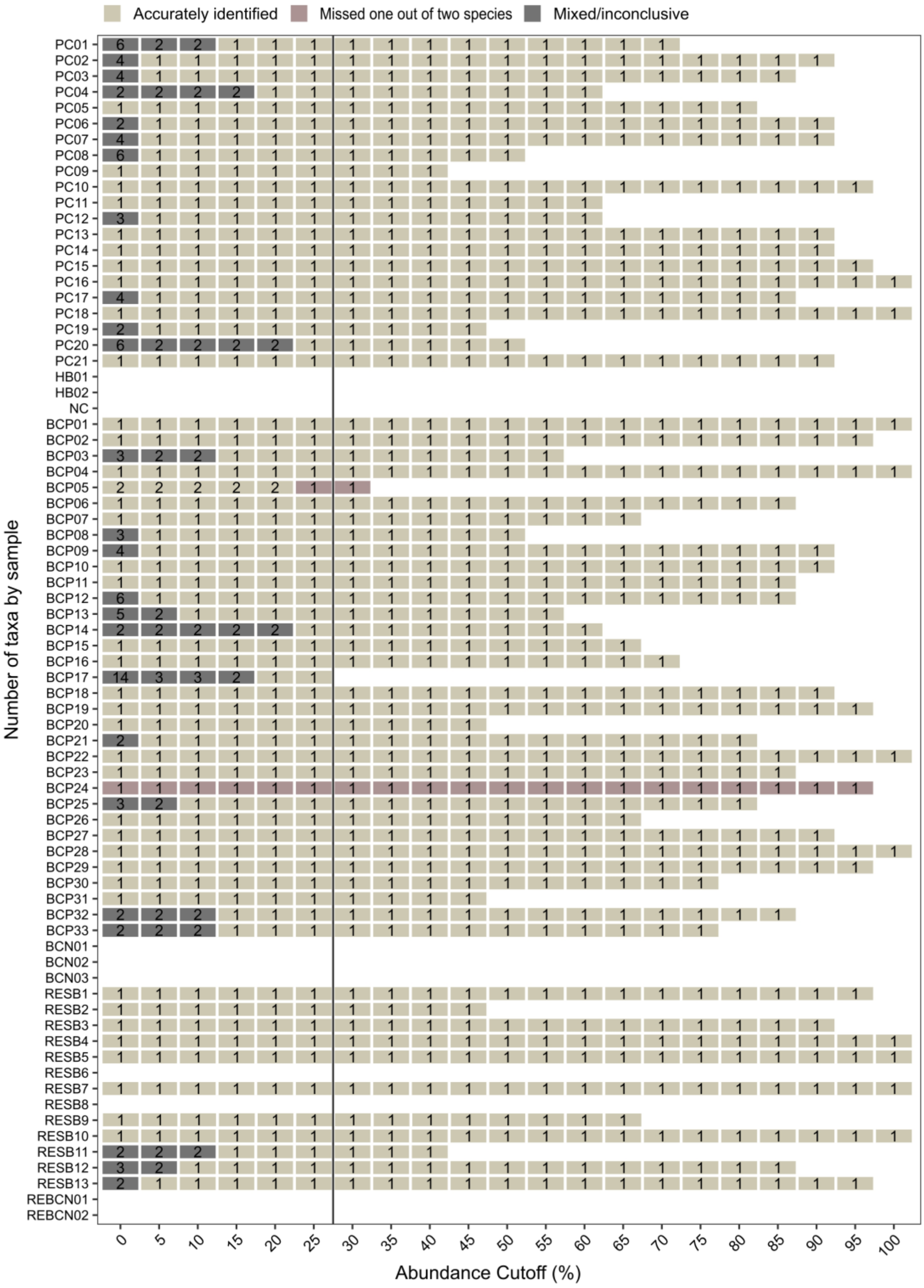

Fig. S1: Abundance cutoff for species identification with MultiSeq AMR. The X-axis represents varying abundance cutoffs and Y-axis all the samples tested in this study. The text within each box indicates the number of species detected for the respective sample and the colour fill represents the accuracy; species accurately identified, missed one of two species, or inconclusive results (see legend). The black line shows the cutoff with highest combined sensitivity and specificity compared to blood culture.

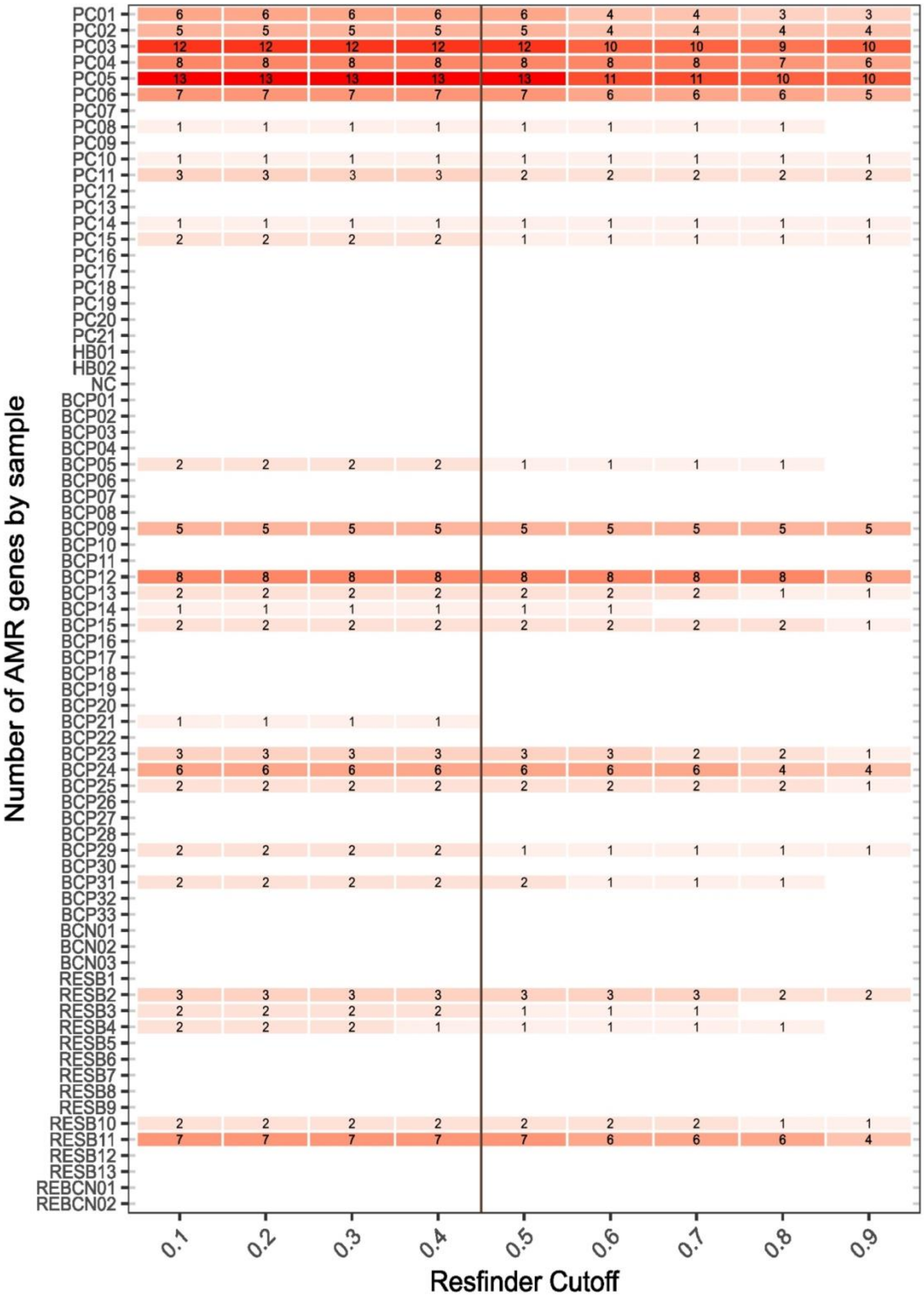

Fig. S2: Resfinder cutoff for AMR gene detection with MultiSeq AMR. The X-axis represents varying Resfinder length and for %ID cutoffs and Y-axis representing all the samples tested in this study (n=75). The text within each box and the fill intensity indicates the number of AMR genes detected for the respective sample. The black line shows the cutoff with the highest combined sensitivity and specificity for detecting AMR determinants compared to whole genome sequencing.
