# Supplementary material for: MultiSeq-AMR: a modular amplicon-sequencing workflow for rapid detection of bloodstream infection and antimicrobial resistance markers
Source: Microb Genom. 2025 Apr 3;11(4):001383. doi: 10.1099/mgen.0.001383 (PMC12452178; doi:10.1099/mgen.0.001383)
Supplement: Supplementary Material 2. [file mgen-11-01383-s004.pdf]

Supplement protocol: MultiSeq-AMR

Consumables

Name	Concentration/units	Commercial source	Comments
MultiSeq primer pools	10 µm per pool	IDT or Eurofins	Check list of primers & pools below
Saponin (Only for REBC samples)	Final concentration 2.25%	Tokyo chemical industry, Cat: S0019	To make a 2.25% w/v solution, dissolve 2.25 grams of Saponin into 50 ml sterile nuclease free H_2O. Mix thoroughly until dissolved. Adjust the volume to 100ml. Sterilise using 0.2 µm filter, and store at 4 °C for up to 3 weeks.
DNase I	300 units/µL	Invitrogen, Cat: 18047019	NA
PCR H20	NA	Qiagen, Cat: 17000-10	NA
DNA binding beads	NA	Aline biosciences, Cat: C-1003-50	NA
Molecular grade ethanol	NA	Sigmaaldrich, Cat: 51976-500ML-F	NA
Qubit dsDNA BR assay Kit	NA	ThermoFisher, Cat: Q32853	NA
Qubit assay tubes	NA	ThermoFisher, Cat: Q32856	NA
PCR master mix	NA	RepliQa HiFi ToughMix, Quantabio, Cat: 95200-500	For ultra rapid PCR amplification and barcoding
Proteinase K	NA	Qiagen, Cat: RP107B-1	
DNA extraction kit	NA	Qiagen, Cat: 50214, with Pathogen Lysis Tubes L, Cat: 19092	Users can choose their own extraction method as long as it is sensitive and robust
DNA/RNA Shield	1x	Zymo research, Cat: R1100-50	NA
Ligation Sequencing Kit	NA	ONT, Cat: SQK-LSK109	NA
PCR Barcoding Expansion Kit	NA	ONT, Cat: EXP-PBC096	NA
NEBNext Ultra II End Repair/dA-Tailing Module	NA	NEB, Cat: E7546L	NA
NEBNext Quick	NA	NEB, Cat: E6056L	NA

Name	Concentration/units	Commercial source	Comments
Ligation Module			
Flow Cell Priming Kit	NA	ONT, Cat: EXP-FLP002	Required for both protocols
Flow Cell Wash Kit	NA	ONT, Cat: EXP-WSH004	Required for both protocols
ONT Flow Cell R9.4.1	NA	ONT, Cat: FLO-MIN106 or FLO-FLG001	Required for both protocols
Magnetic rack/plate holder	NA	ThermoFisher, Cat: 12331D and 12321D	NA
PCR plate 96 well	NA	Merck, Cat: BR781378	NA
1.5 ml DNA LoBind tubes	NA	Eppendorf, Cat: 0030108051-250EA	Use LoBind tubes
2 ml round bottom tubes	NA	Starlab, Cat: S1620-2700-C or Eppendorf, Cat: 0030120094	Avoid using LoBind tubes
Pipettes and pipette tips P2, P10, P20, P100, P200, P1000, Multichannel	NA	Any	NA
Centrifuge /Microplate centrifuge	NA	Any	NA
Heating block for 1.5/2 mL tubes	NA	Any	NA
Sequencing device	NA	Flongle, MinION Mk1B, GridION	NA
Device for live basecalling	NA	GridION or A powerful laptop	A laptop with minimum 8 Gb GPU (RTX 3060 or higher), 16 GB Ram, 1 Tb M.2 NVMe SSD

Disclaimer: Some parts of the following protocol has been copied directly from the manufacturer's official website or from the kit manual. Therefore, all the proprietary information mentioned below remains the sole property of the kit's manufacturer.

Before sample preparation

It is important to divide all reagents into smaller aliquots to avoid multiple freeze-thaw cycles and reduce the likelihood of cross-contamination. Thaw all the reagents at room temperature or on ice as per manufacturers recommendation.

This protocol can be used on BD BACTEC (Becton, Dickinson and Company, NJ, USA) or any blood culture (FPBC) samples flagged positive with an automated culture system. Protocol M-15 mNGS can also be applied to rapid culture-enriched blood samples (REBC; for example,

1-10 mL patient blood samples incubated for 6 hours in BD BACTEC media bottles), once the enriched samples have at least 103 CFU/mL bacterial concentration. FPBC samples can be used directly for chemical host DNA depletion (CHDD); however, REBC samples need to be pre-processed to concentrate the sample before continuing with this protocol. It is crucial to use fresh REBC samples for CHDD, as frozen or improperly stored samples can lead to the lysis of bacterial cells. This may lead to a considerable loss of bacterial DNA during CHDD.

Pre-processing REBC samples

1. Transfer 1.5 mL REBC samples carefully to a 2 mL round bottom microcentrifuge tube and centrifuge at 8,000 xg for 5 minutes.

   Note* It is important to use round/wide bottom 2 mL microcentrifuge tubes to minimise bacterial cell loss while aspirating the supernatant during the initial washing steps.

2. Slowly aspirate the supernatant and resuspend the pellet in 800 µL 2.25% saponin solution.
3. Add 1 µL DNase I (300 units/µL) to the sample and incubate at 25°C in a shaking heating block for 10 minutes at 800 rpm.
4. Centrifuge the tube at 10,000 xg for 5 minutes, aspirate the supernatant carefully and resuspend the pellet in 1x DNA/RNA Shield according to the input volume for the DNA extraction kit (for example, 400 µL for QIAamp UCP kit).

DNA extraction (Mechanical Pre-lysis Protocol for whole blood)

400 µL FPBC or REBC samples in 1x DNA/RNA Shield can be extracted using QIAamp UCP Pathogen Mini kit with Pathogen Lysis Tubes L following manufacturer's instructions. Following extraction and purification steps, elute the DNA in 50 µL Buffer AVE and then proceed to the rapid PCR barcoding step.

Note* Alternative bacterial cell lysis and DNA extraction methods (e.g., automated or faster) can also be used, however, for the rapid enrichment method, it is important that the users test the efficiency in lysing tough bacterial cells and DNA recovery rate/analytical sensitivity for low abundant samples before proceeding.

Protocol for mechanical pre lysis (Pathogen Lysis Tubes L) with QIAamp UCP Pathogen Mini kit can be accessed from the link below.

https://www.qiagen.com/us/resources/download.aspx?id=0930379e-a52a-475a-acc5-d944701dbaeb&lang=en

Rapid PCR barcoding of AMR and species-specific targets

Combine 100 µM stock primers (forward and reverse) in equal volumes (e.g., 10 µL each) for each primer according to the following combinations for each pool:

Pool 1	Pool 2	Pool 3	Pool 4	Pool 5	Pool 6	Pool 7	16S + 28S

blaNDM	*blaSHV*	*blaTEM*	*blaPER*	*cmlA*	*ermA*	*dfrA1*	16S rRNA
blaKPC	*mcr-1*	*blaOXA-9*	*blaCMY1/MOX*	*sul1*	*ermB*	*dfrA12*	NL1-NL4
blaIMP	*mcr-2*	*blaOXA-23*	*blaCMY-2*	*sul2*	*aph(3')-III*	*dfrA7/17*	NA
blaVIM	*vanA*	*blaOXA-24*	*AAC(6')-Ib*	*ant(2")-I*	*aph(3')-IIa*	*str*	NA
blaGES	*vanB*	*blaOXA-58*	*qnrB*	*aac(3)-II*	*aph(3')-Ia*	*catA1*	NA
blaOXA-48	*mecA*	*blaFOX*	*qnrS*	*aac(3)-IV*	*aadA1*	*catB7*	NA
blaCTX-Group-M-All	*mecC*	*blaACC*	*qepA*	*mphA*	*aadA2*	*oqxA*	NA
Pool 1 to Pool 7 and 16S+28S tested in this study							

Pool 8	Pool 9	Pool 10	Pool 11	Pool 12	Pool 13
blaOXA-1	*blaGIM*	*gyrA*	*msrA*	*parC*	*tet(A)*
blaOXA-7	*blaSPM*	*ermC*	*msrC*	*oqxB*	*tet(B)*
blaOXA-50	*blaVEB*	*ermF*	*msrD*	*catA2*	*tet(C)*
blaOXA-51	*blaBEL*	*ermG*	*msrE*	*sul3*	*blaDHA*
qnrA	*mcr-3*	*ermT*	*cmlB*	*mefA*	*blaACT/MIR*
qnrC	*mcr-4*	*mphB*	*rmtB*	*mefB*	*aph(3')-Ib*
qnrD	*mcr-5*	*mphE*	*armA*	*mefE*	*dfrA5*
Pool 8 to Pool 14 not tested in this study					

Dilute the 100 µM pools at 1:10 ratio in molecular grade nuclease free water to create stocks of 10 µM primer pools. Primers are used at a final concentration of 50 nanomolar (nM) per primer in MultiSeq-AMR protocol. AMR primers are combined to target up to 7 AMR targets per pool (can be extended further). The primer targets and/or pools are modular and can be customised/expanded (from 8 pools, and 12 samples to 12 pools, 8 samples per PCR plate) according to requirements.

1. Combine the following components using a multichannel pipette and dispense into a 0.2 mL PCR plate. For example, for Sample1 using Barcode1: (Up to 96 samples, can be processed per batch).

Note* As an alternative, liquid handlers (conventional or acoustic) can be used for 0.5x, 0.25x volume and for faster aliquoting.

Reagent (µL) 1x	Pool 1	Pool 2	Pool 3	Pool 4	Pool 5	Pool 6	Pool 7	16S + 28S
RepliQa HiFi	10	10	10	10	10	10	10	10

ToughMix (2x)								
MultiSeq primers (10 µM)	1.4	1.4	1.4	1.4	1.4	1.4	1.4	0.4
PCR barcodes (10 µM)	1	1	1	1	1	1	1	1
Purified DNA	3	3	3	3	3	3	3	3
PCR H_2O	4.6	4.6	4.6	4.6	4.6	4.6	4.6	5.6
Total	20	20	20	20	20	20	20	20

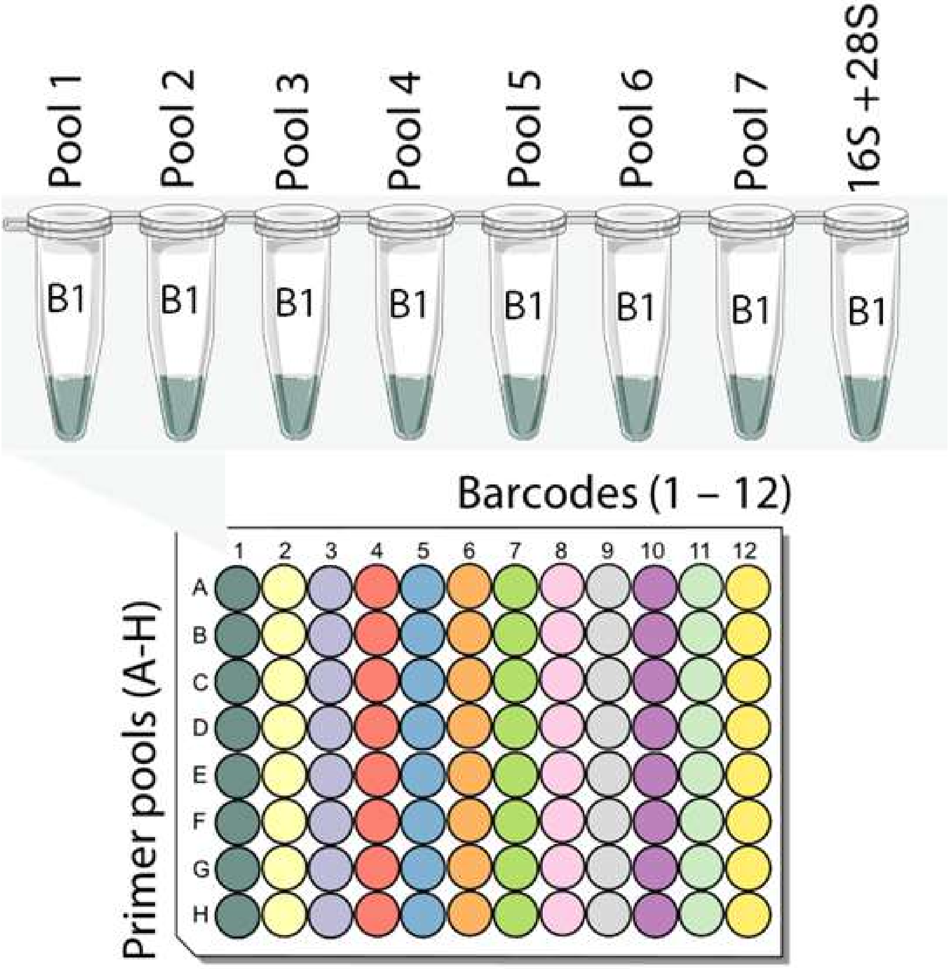

2. Seal the plate tightly, spin and perform PCR with the following conditions with the heated lid on.

Step	Primers active	Temp (°C)	Time (seconds)	Cycles
Initial denaturation	None	98	120	1

Denaturation	AMR/species	98	10	5-7*
Annealing		58	10	
Extension		68	15	
Denaturation	AMR/species	98	10	5-7*
Annealing		57	10	
Extension		68	15	
Denaturation	AMR/species	98	10	5-7*
Annealing		56	10	
Extension		68	15	
Denaturation	AMR/species	98	10	5-7*
Annealing		55	10	
Extension		68	15	
Denaturation	Barcode	98	10	10-12*
Annealing		62	10	
Extension		68	15	
Final extension	None	68	60	1

Note* For FPBC samples, perform (5x4 = 20 + 10) a total of 30 PCR cycles. For REBC, perform (7x4 = 28 + 12) 40 PCR cycles overall.

3. Following PCR, spin down the materials by performing a quick spin and combine pools 1 through 8 using a multichannel pipette. Take 5 µL PCR product from pools 1 to 7 and 10 µL (half) from the 16S/28S pool.
4. Resuspend the DNA binding beads (e.g., AMPure XP or Aline biosciences) by vortexing.

   Note* DNA binding beads can be aliquoted in PCR strips. This can be done upon receiving all the reagents, so these steps do not take addition time when processing the samples.

5. Add 2 µL of 20 mg/mL proteinase K to each sample tube using a multichannel pipette.

Note* For this 20 mg/mL proteinase K needs to be aliquoted in PCR strips. This can be done upon receiving all the reagents, so these steps do not take addition time when processing the samples.

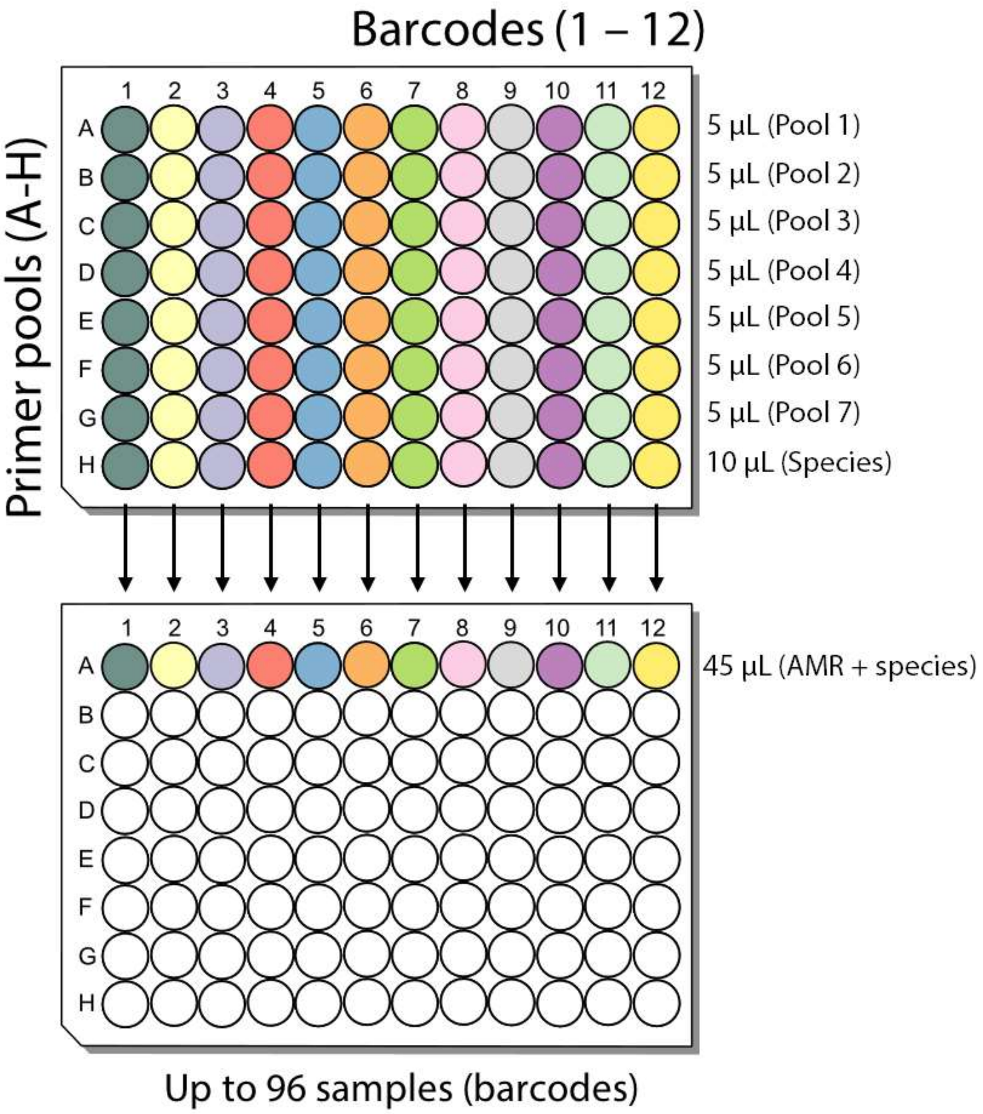

6. Similarly, add 23 µL bead suspension (approximately 0.5x) per sample using a multichannel pipette and mix by pipetting up and down.
7. Incubate the sample for 5 minutes at room temperature.
8. Pellet the sample on a magnetic stand until the supernatant is clear and colourless. Pipette off the supernatant by keeping the tube on the magnet.
9. Keep the plate on the magnetic stand and wash the beads with 200 µL of freshly prepared 70% ethanol without disturbing the pellet. Aspirate the ethanol carefully without touching the pellet using a multichannel pipette and discard.
10. Repeat the previous ethanol washing step.
11. Pipette off any residual ethanol by keeping the tube on the magnet. Allow to air dry for about ~30 seconds, but do not dry the pellet to the point of cracking.
12. Remove the plate from the magnetic stand and resuspend pellet in 25 µL nuclease-free water. Incubate for 2 min at room temperature.

13. Pellet the beads on a magnet until the eluate is clear and colourless (at least for one minute).
14. Remove and retain 20 µL of eluate into a clean PCR plate.
15. Quantify 1 µL barcoded DNA from each sample using a Qubit fluorometer.

Ligation sequencing

End prep

1. Pool up to 96 samples in a 0.2 mL PCR tube, ensuring the total DNA amount is exactly 200 ng (or approximately 400 fmol, assuming a fragment size of 0.75 kb) in a volume of 49 µl, using the following procedure. The example below is showing pooling procedure for 12 samples.

Samples	Barcode	DNA (ng)	Volume (µl)
Sample 1	01	16.6	4.08
Sample 2	02	16.6	4.08
Sample 3	03	16.6	4.08
Sample 4	04	16.6	4.08
Sample 5	05	16.6	4.08
Sample 6	06	16.6	4.08
Sample 7	07	16.6	4.08
Sample 8	08	16.6	4.08
Sample 9	09	16.6	4.08
Sample 10	10	16.6	4.08
Sample 11	11	16.6	4.08
Sample 12	12	16.6	4.08
Total		200	49

2. Now combine the following reagents in a 0.2 mL PCR tube.

Reagent	Volume (µL)
DNA control sample (DCS)	1
Ultra II End-prep Reaction Buffer	7
Ultra II End-prep Enzyme Mix	3
Pooled DNA (200 ng)	49
Total	60

3. Mix the samples gently by flicking the tube multiple times, and spin down.
4. Incubate the sample at 20°C in a thermal cycler for 5 minutes with heated lid option turned off.
5. Next, incubate sample again at 65°C for 5 minutes with heated lid on.

Native barcode ligation and cleanup

6. Combine the following reagents in a 0.2 mL PCR tube.

Reagent	Volume (µL)
End prepped DNA	60
Adapter Mix (AMX or AMX-F)	5
Ligation Buffer (LNB)	25
Quick T4 DNA Ligase	10
Total	100

7. Flick to mix the sample multiple times, spin and incubate the reaction mixture for 10 minutes at 20 °C.
8. Resuspend the DNA binding beads (e.g., AMPure XP or Aline biosciences) by vortexing.
9. Add 100 µL adapter ligated DNA sample to 50 µL (0.5x) of resuspended DNA binding beads in a 1.5 mL Eppendorf DNA LoBind tube and mix by pipetting.
10. Incubate the mixture for 5 minutes at room temperature.
11. Spin down the sample and pellet on a magnetic rack until the supernatant is clear and colourless. Keep the tube on the magnet, and carefully pipette off the supernatant.
12. Wash the beads by adding 250 µl Short Fragment Buffer (SFB).
13. Repeat the previous SFB washing step.
14. Spin down and place the tube back on the magnetic rack. Pipette off any residual SFB. Allow the tube to dry for ~30 seconds, but do not dry the pellet to the point of cracking.
15. Remove the tube from the magnetic rack and resuspend the pellet in 17 µL Elution Buffer (EB).
16. Spin down briefly and incubate the mixture for 2 minutes at room temperature.
17. Pellet the beads on a magnetic rack until the eluate is clear and colourless, for at least 1 minute.
18. Remove and retain 15 µL of the eluate (EB) containing the DNA library into a clean 1.5 mL Eppendorf DNA LoBind tube.

19. For MinION flow cell, load approximately 25 ng (51.36 fmol; considering 0.75kb) of final prepared library onto the MinION flow cell following manufacturers recommendation. Alternatively, if processing a few samples per batch, load 10 ng (20.54 fmol; considering 0.75kb) of final prepared library onto the Flongle flow cell.

Note* The typical library size after this stage is around 0.75 kb. However, users can verify this by running the library on a gel or TapeStation for a few batches. Once the average expected library size is determined (sample or site-wise), there will be no need to repeat this for every batch of samples.

For loading sequencing library or washing flow cells, follow the manufacturers recommended protocols.

Priming and loading sequencing libraries

https://nanoporetech.com/document/rapid-barcoding-sequencing-sqk-rbk004#priming-and-loading-the-sp

https://community.nanoporetech.com/nanopore_learning/lessons/priming-and-loading-your-flow-cell

Washing flow cells for re-use

https://nanoporetech.com/document/flow-cell-wash-kit-exp-wsh004
